# Overview of harm reduction in prisons in seven European countries

**DOI:** 10.1186/s12954-016-0118-x

**Published:** 2016-10-07

**Authors:** Gen Sander, Alessio Scandurra, Anhelita Kamenska, Catherine MacNamara, Christina Kalpaki, Cristina Fernandez Bessa, Gemma Nicolás Laso, Grazia Parisi, Lorraine Varley, Marcin Wolny, Maria Moudatsou, Nuno Henrique Pontes, Patricia Mannix-McNamara, Sandro Libianchi, Tzanetos Antypas

**Affiliations:** 1 Harm Reduction International, London, UK; 2 Associazione Antigone, Rome, Italy; 3 Latvian Centre for Human Rights, Riga, Latvia; 4 Education Centre, Midlands Prison, Portlaoise, Ireland; 5 PRAKSIS, Athens, Greece; 6 University of Barcelona, Barcelona, Spain; 7 UL Hospitals Group, Limerick, Ireland; 8 Helsinki Foundation For Human Rights, Warsaw, Poland; 9 CIES-IUL/ISCTE-IUL Instituto Universitário de Lisboa, Lisboa, Portugal; 10 Research Centre for Education and Professional Practice, University of Limerick, Limerick, Ireland; 11 Medical Services in Rebibbia Prison (Rome) and Coordinamento Nazionale degli Operatori per la Salute nelle Carceri Italiane, Rome, Italy

**Keywords:** Prison, Prisoner, Prison health, Harm reduction, Opioid substitution therapy, Needle and syringe programme, HIV, Hepatitis C, Human rights

## Abstract

While the last decade has seen a growth of support for harm reduction around the world, the availability and accessibility of quality harm reduction services in prison settings is uneven and continues to be inadequate compared to the progress achieved in the broader community. This article provides a brief overview of harm reduction in prisons in Catalonia (Spain), Greece, Ireland, Italy, Latvia, Poland, and Portugal. While each country provides a wide range of harm reduction services in the broader community, the majority fail to provide these same services or the same quality of these services, in prison settings, in clear violation of international human rights law and minimum standards on the treatment of prisoners. Where harm reduction services have been available and easily accessible in prison settings for some time, better health outcomes have been observed, including significantly reduced rates of HIV and HCV incidence. While the provision of harm reduction in each of these countries’ prisons varies considerably, certain key themes and lessons can be distilled, including around features of an enabling environment for harm reduction, resource allocation, collection of disaggregated data, and accessibility of services.

## Background

The last decade has seen a growth of support for harm reduction around the world. Harm reduction programmes are now operating at some level in more than half of the world’s 158 countries where injecting drug use has been documented; in other words, it is now a majority response to drugs [1]. In Europe, the situation appears even rosier. All 28 European Union (EU) member states have expressed explicit support for harm reduction in national policies and have made needle and syringe programmes (NSPs) and opioid substitution therapy (OST) operational at the national level [2]. While this is a wonderful achievement, as always, there is plenty of room for improvement. Although crucial harm reduction services are now available in a majority of countries, including in all EU member states, in many countries, programmes are not operating to scale or meeting the needs of the population they reach. Across much of the region, harm reduction remains unavailable to one particularly key population: prisoners.^1^

Like in all other regions of the world, repressive drug laws across Europe have resulted in people who use drugs being overrepresented in the prison system. Currently, between 10 and 25 % of all sentenced prisoners in Europe are incarcerated for crimes related to the use, possession or supply of illicit drugs [3]. In 2013, an estimated 230,000 supply offences and 1.1 million drug use or possession offences were reported [3]. Prisoners report higher lifetime rates of drug use than the broader community, along with more harmful patterns of use. Studies show that between 5 and 38 % of prisoners in Europe admit that they have ever injected drugs [3], while between 2 and 31 % of prisoners in the EU, depending on the country, are reported to have ever injected drugs while in prison [4]. Although they may inject less frequently, prisoners are much more likely to share injecting equipment and with a greater number of people [4].

For these and other reasons, including overcrowding, poor sanitation and inadequate health care, prisons represent high-risk environments for the transmission of human immunodeficiency virus (HIV), hepatitis C virus (HCV) and tuberculosis (TB). This makes them important settings for the provision of evidence-based harm reduction services, including NSPs, OST, antiretroviral therapy (ART), opioid antagonists (e.g. naloxone), condoms and education and information on harm reduction.

This is also a human rights issue. Prisoners retain their right to the highest attainable standard of health while incarcerated, which includes a right to preventive health services [5] and harm reduction services [6]. Denial of these services in prison settings has also been found to contribute to, or even constitute, conditions that meet the threshold of ill treatment [7]. Echoing widely accepted minimum standards and guidelines on prison health and prisoners’ rights, such as the United Nations Basic Principles for the Treatment of Prisoners [8], many within the UN system have now confirmed that providing harm reduction services to the general public but not to prisoners is a flagrant violation of international human rights law [9].

The current EU Drugs Strategy specifically identifies scaling up the development, availability and coverage of harm reduction measures in prison settings as a priority, with the aim of achieving a quality of care equivalent to that provided in the community and in accordance with the right to health and human dignity [10]. Throughout the EU, however, the introduction or scale up of harm reduction measures in prisons is uneven and continues to be inadequate compared to the progress achieved outside of prisons.

This article will present a brief overview of the harm reduction situation in prisons in seven European countries: Spain (Catalonia), Greece, Ireland, Italy, Latvia, Poland, and Portugal. A short description of the relevant legal and policy context, available harm reduction services in the broader community and prison health more generally are also included to provide a better idea of the environment in which harm reduction services are—or are not—being provided in prison settings. This overview is part of an EU co-funded project on HIV, HCV, TB and harm reduction in prisons being led by Harm Reduction International and is derived from one of the project’s mapping components consisting a detailed report on HIV, HCV, TB and harm reduction in prisons, as well as prison monitoring practice on these issues in each country, prepared by local researchers.^2^ While the provision of harm reduction in each of these countries’ prisons varies considerably, certain key themes and lessons can be distilled, including around features of an enabling environment for harm reduction, resource allocation, collection of disaggregated data and accessibility of services. These will be briefly discussed in the ‘Conclusions’ section in the hopes that they can be instructive to countries wishing to scale up harm reduction in their prisons or to anyone advocating for harm reduction in prisons.

## Catalonia (Spain)^3^

### Legal and policy context

Spain is made up of two autonomous cities and 17 autonomous communities, one of which is Catalonia. The country’s constitution enshrines the right to health for all [11], and national laws and standards further protect prisoners’ health rights [12]. Both the Spanish and Catalan health systems are revered due to their extensive coverage, but like in many other countries in the region, the economic crisis has led to increasing privatisation and budget cuts, all of which have impacted the entire population’s access to health care, prisoners not excluded.

While health care and many other issues are decentralised to some degree, the central government has exclusive jurisdiction over criminal law. Drug use and possession for personal use in the country do not constitute a criminal offence, although drug dealing and trafficking carry heavy penalties. The Penal Code has been amended a few times to introduce, among other things, rehabilitative mechanisms to support people who use drugs, such as conditional suspensions of prison penalties of 5 years or less if the person is following a detoxification programme [13].

Catalonia has certainly created an enabling environment for the implementation of harm reduction, including in prisons. The 2009–2016 National Drug Strategy prioritises drug-related harm reduction and harm reduction services such as NSPs, OST, drug consumption rooms and overdose prevention programmes, including the distribution of naloxone, have been widely available throughout the region since the 1980s.

### Prison health

In November 2014, Catalonia transferred authority for prison health from the Department of Justice to the Department of Health, making it the first and only autonomous community in Spain to have done this. The prison health system is now fully integrated within the broader public health system, helping to ensure a standard of care for prisoners that is equivalent to that available to the broader community. The Catalan Health Department will also soon be implementing a new project called ‘contact nurse’, whereby some nurses will have as their main responsibility helping to ensure that prisoners enjoy a continuity of care following their release [14].

There are currently 11 prisons and one penitentiary hospital in Catalonia. The prison population as of 31 December 2015 was 8810 [15],^4^ 20.02 % of which were incarcerated on drug trafficking charges [15]. While no official data on drug consumption or injection within prisons is available, official data on HIV, HCV and TB in Catalan prisons are published online and updated every 3 months. In 2014, 92 % of the prison population underwent voluntary screening for HIV, viral hepatitis and tuberculosis [16]. Voluntary tests are offered every 6 months within Catalan prisons, and treatment is initiated immediately following any detection of disease. In 2014, 1427 prisoners were living with HCV and 519 with HIV, approximately 73.4 % of which contracted the virus through sharing injecting equipment [17]. While prevalence rates have been steadily declining in Catalan prisons—for example, HCV prevalence has decreased from 43.6 to 19.2 % since 2004 [18]—they continue to be much higher than in the broader Catalan community. Specifically, TB rates are 10 times higher, HCV rates 7.2 times higher, and HIV rates 20–28 times higher. Unfortunately, none of the official statistics on communicable diseases in prisons are disaggregated on any of the prohibited grounds of discrimination,^5^ making it difficult to identify any glaring disparities.

### Harm reduction in prisons

The first harm reduction initiatives in Catalan prisons were introduced in the 1980s and involved raising awareness about safer injecting practices and distributing hygienic packs containing brushes, blades and bleach. OST was first made available in some prisons in 1993, amidst much controversy and scepticism [16]. Its uptake and success, however, led to its expansion. Today, OST is now available across all Catalan prisons and it is considered a low-threshold programme, meaning that very few conditions must be met in order to register. In 2014, 1300 prisoners were registered in the OST programme, 200 of which enrolled that year [16]. This number has been decreasing over the years, however, likely due to a reduction in injecting drug use in the community. In 2014, only 7 % of new prisoners self-identified as injecting drug users [16].

Due to serious opposition from the prison workers’ union for many years, NSPs did not become available in Catalan prisons until much later. Today, however, NSPs are provided in all Catalan prisons except for the *Centre Penitenciari d’Homes de Barcelona*, which holds mainly pre-trial detainees. Most doctors and nurses, however, believe that, in accordance with international guidance, they should also be provided there. NSPs are situated in prison health centres and are run by prison health care staff members. Prisoners who are registered in the programme are able to go to the health centre to collect sterile injecting equipment without needing to provide an explanation to prison guards or others and are also allowed to keep injecting equipment in their cells. For safety reasons, bowls are not included in the injecting equipment package that is available to prisoners, which means that the programme is not as complete as the one provided in the broader community. This, alongside concerns about confidentiality, may partially explain why only 5–6 % of people who inject drugs in prisons registered in the needle and syringe programme [16].

Condoms are generously distributed within all Catalan prisons. They are included in hygiene packs that are regularly supplied to prisoners and are provided for conjugal visits and more generally upon request.

Finally, a programme for safer tattooing targeted at youth was piloted in 2010–2011. A professional tattoo artist was made available within the prison, and information on safer tattooing was provided. Tattoo restrictions, however—and in particular those associated with gang symbols—made the programme unappealing to prisoners and it was ultimately discontinued due to low interest.

## Greece^6^

### Legal and policy context

The right to health of all citizens is protected in Greece’s national law. As is often case, however, there is a clear disjunction between law and practice, particularly since the financial crisis and austerity measures triggered the implosion of the country’s already weak social welfare system. While this has been felt across all sectors, the health sector has suffered the most, with serious cuts to financial and human resources resulting in fewer and poorer quality services across the country, including in prisons.

Although drug use, possession and trafficking have been criminalised and punished by imprisonment in Greece by various laws since 1919, the most recent drug law (4139/13) is based on the philosophy that people who use drugs should not be treated as criminals, but rather as ‘patients’ [19]. Despite these changes, as of 1 January 2015, 25 % of the prison population was being held on drug charges [20].

Harm reduction service provision was scaled up in Greece following an outbreak of HIV among people who inject drugs in Athens in 2011. Today, low-threshold services implement a broad range of harm reduction interventions, mostly in the Greater Athens area and Thessaloniki, including NSPs, OST, overdose prevention, condom provision and printed health education and information materials on safer drug use and available services [21]. Today, OST is available in most cities and provided by OKANA, the only agent legally permitted to do so. Waiting times are generally low, apart from in and around Athens, where people can wait up to 3.5 years to access the service. In October 2013, a drug consumption room was opened as a pilot project but, due to delays in establishing a valid legal basis for its operation, it was suspended in July 2014 [22].

### Prison health

Prison health, which is under the authority of the Ministry of Justice, has also been affected by the financial crisis. Not only have prisons themselves experienced serious cutbacks, including to health care staff, but civil society organisations have also had their budgets slashed, resulting in many having to withdraw the health services they were providing in prison settings. Only one prison in Greece has a hospital onsite, while all other prisons are visited by external doctors on specific days and during specific times. Effectively, this means that prisoners have no direct access to health services, which is a violation of the principle of equivalence.

As of January 2015, there were 11,798 prisoners, including pre-trial detainees, in 35 different institutions [23].^7^ Very few studies have been done on health in prisons, and national data on health is not disaggregated by legal status. At the prison level, there is no standardised system in place to collect data on prisoner health, although the development of an electronic database is now under consideration. As a result, there is currently very little or no data on prisoners who use or inject drugs, or who are living with HIV, HCV and/or TB, which is problematic and makes monitoring and evaluating progress very difficult. It is safe to assume, however, that prevalence rates remain higher than those in the community and that injecting drug use does take place in prisons.

The European Committee for the Prevention of Torture and Inhuman or Degrading Treatment and the Greek Ombudsman have found on several occasions that lack of health care services and staff, poor sanitary conditions, insufficient infrastructure and overcrowding constitute an unhealthy and dangerous environment for prisoners, in certain cases amounting to inhuman or degrading treatment [24].

### Harm reduction in prisons

There is still no comprehensive, nationwide approach to harm reduction in the Greek prison system. The law provides that any prisoner wishing to attend a rehabilitation programme can apply for release and steps are taken to facilitate their integration into this programme [25]. For those who stay in prison, however, most options available to them are known as ‘therapeutic programmes’, i.e. those provided by 18+ and KETHEA. They are drug-free, focused on rehabilitation, therapy and social reintegration and often restricted to those that meet quite strict criteria, including having no history of mental illness and the ability to understand Greek, despite more than half the country’s prison population consisting of non-Greek nationals [26]. Civil society has reported very high mortality rates—up to 95 %—in the first 2 weeks following the release of prisoners participating in these therapeutic programmes [24].

NSPs have never been provided in Greek prisons, and OST only became available in 2014 in St. Stephen prison and Korydallos Judicial prison. Run by national non-governmental organisations (NGOs), after months of operation, only 80 prisoners were accessing the service and over 400 requests were on hold. According to recent reports, OST may be scaled up in Greek prisons in the future as efforts to include this service in OKANA’s basic budget have apparently been underway.

## Ireland^8^

### Legal and policy context

Although the right to health has not been explicitly incorporated into national law in Ireland, international treaties protecting health rights have been ratified and everyone living in the Republic, including prisoners, is entitled to receive health care through the public health care system, which is managed by the Health Service Executive.

Drugs use, possession and supply are criminalised in Ireland. In terms of use, only prepared opium is prohibited and punished by imprisonment and/or a fine. Possession of cannabis carries with it a fine for the first couple of offences, whereas possession of other drugs and the supply of drugs generally immediately result in imprisonment. Following each conviction, courts have the option of referring the ‘offender’ to medical treatment or drug treatment courts instead of imposing the penalty, although this appears to happen infrequently [27].

There is broad support for harm reduction in Irish health and drug-related policies and strategies, which have led to the implementation of some important harm reduction services in the general community. NSPs have been operating in the country since 1989 and are currently provided via 24 static sites, 14 outreach sites and 129 community-based pharmacies [28]. OST was introduced in 1992 and is now widely available throughout the country, including to pregnant opiate users who are entitled to immediate access, and through specific initiatives targeting under-18s. OST is provided by treatment centres, specialised general practitioners and in prisons. In 2013, in an effort to improve the quality of the service, new guidelines for prescribing methadone during pregnancy were issued and the first national clinical guidelines are currently under review [29].

With one person dying from an overdose every day, there have been some developments in the last year to reform the country’s drug policies, including decriminalising possession of small amounts of drugs and making more harm reduction services available. In 2015, the Health Service Executive rolled out a ‘demonstration project’ that involved training non-medical staff, such as care workers, family and peers, in the administration of naloxone, which has been hailed as an important step towards making naloxone more widely available to people who use drugs [30]. The current Minister of State responsible for Ireland’s Drug Strategy, Aodhán Ó Ríordáin, also recently announced that the country will be opening drug consumption rooms in four Irish cities in 2016 [31].

### Prison health

As a result of the structural reforms imposed by the EU/International Monetary Fund financial assistance package, the Irish Prison Service experienced budget cuts of over €72 million between 2008 and 2014, despite an increase in the number of prisoners [32, 33].

Prisoner health in Ireland is under the authority of the Irish Prison Service, as opposed to the Ministry of Health. Finalised in 2011, the health care standards for the Irish Prison Service, which include standards on drug treatment and communicable diseases, aim to provide prisoners with access to the same quality and range of health services as those available to the broader community [34]. This, however, has not been the case in practice. Health care in Irish prisons has been described as disconnected from the national health care service and in a state of crisis [35].

As of July 2015, there were 3771 individuals imprisoned in 14 institutions operated by the Irish Prison Service.^9^ In 2013, nearly 16 % of the prison population were serving sentences for drug crimes [33]. The prevalence of drug use within Irish prisons is reported to be much higher than in the general community, with lifetime prevalence of injecting drug use among prisoners recorded at 25 % [36]. Data on the incidence of HIV, HCV and TB in Ireland is not disaggregated by legal status, so there is a serious shortage of information on these diseases in Irish prisons. Some independent research has been done, however, identifying HIV and HCV prevalence at 2 and 13 %, respectively, in Irish prisons [36]. In recent years, high incidences of TB outbreaks associated with multi-drug resistance and HIV co-infection have been observed in prison populations [37].

### Harm reduction in prisons

Ireland’s National Drugs Strategy 2009–2016 includes a specific focus on prisons, and action 43 refers to the need for ‘seamless provision of [drug] treatment services’ between the prison and the broader community. As of 2014, OST was available in all 14 prisons and access to this service was considered high [2]. Quality, however, varies by institution. According to the Irish Prison Service, OST provision at Mountjoy prison is currently equivalent to OST provision in the broader community. It includes six specialist nurses who have a distinct role with regard to drug dependence assessment, treatment planning, as well as delivery and evaluation of care. It also consists of a clinical drug dependence team that is made up of all disciplines engaged in drug treatment services. While there are plans to make this standard of OST available in all other prisons, particularly those where demand for OST in high [38], the standard of OST available in other prisons confirms there is a long way to go. During its visit to Ireland 2014, the European Committee for the Prevention of Torture learned that the Limerick Prison doctor refused to prescribe methadone and that the ‘methadone doctor’ only visited twice a week, resulting in a haphazard and incomplete treatment programme [35]. Other known harm reduction services available in Irish prisons include specialised counselling or psychiatry for drug dependence, as well as community-run counselling. NSPs and naloxone are not currently provided.

## Italy^10^

### Legal and policy context

Italy’s 1948 constitution explicitly recognises health as a fundamental human right, and the Italian National Health Service was established in 1978 to facilitate universal access to a uniform level of health care that is free at the point of use. Planning and delivery of health services, however, has been the responsibility of autonomous regional governments since 2011, which has led to the creation of 21 distinct health care systems with wide variations in coverage of services, quality of care and, ultimately, health outcomes.

Drug laws in the country in the past 40 years have fluctuated between hard line blanket prohibition approaches and more reasonable regulatory approaches, depending on the political climate. Today, possession, acquisition and import for personal consumption remain prohibited but only attract administrative sanctions, and fixed quantities are no longer applied to determine the difference between use and traffic. Production and trafficking carry with them a penalty of imprisonment and a fine according to quantity and drug type. Offenders who use drugs have the option of applying to join a drug treatment programme as an alternative to incarceration, although a judge makes the final decision on this [39].

Although Italy’s drug policy focuses more on prevention and reduction of drug use than on harm reduction, some harm reduction services are available in the country. OST (methadone and buprenorphine) and NSPs are both available in the broader community. These are predominantly provided in the northern and central regions, however, which have the greatest urban densities and where the highest numbers of people who use drugs are reported to reside. These services are delivered through fixed sites, mobile units, outreach programmes and needle and syringe dispensing machines [40].

### Prison health

In 2008, prison health became the responsibility of the Ministry of Health. While this was a milestone for the protection of prisoners’ health, the regionalisation of the health service has led to discrepancies in service provision and quality, as well as prisoner health outcomes, depending on the location of the prison. Prisoners have raised complaints about the lack of preventive health care and delays in diagnoses.

As of 31 January 2015, there were 206 prisons in Italy holding 53,889 prisoners.^11^ Although there have been improvements regarding overcrowding in the last few years, with the official capacity at 49,943, there are still 108 prisoners for every 100 available spaces. As of 31 December 2014, there were 18,946 prisoners incarcerated for drug offences.

Data on HIV, HCV and TB in prisons is scarce. Each region is responsible for data collection; some regions, however, do not gather data systematically, and if it is gathered, it is not shared with the Ministry of Health or Justice. The Ministry of Health and the Regional Health Agency of Tuscany collected the most recent data from 58 prisons spread over six regions. Of 15,751 prisoners tested, 7.4 % were found to be living with HCV, 2 % with HIV and 0.6 % with TB [41]. These figures are much higher than those in the broader community, particularly with regard to HCV, which is three times higher among prisoners [41]. This data was also disaggregated by sexual identity, age and ethnic group, which revealed significant discrepancies. Transgendered prisoners, for example, were found to be considerably more likely to be living with HIV and/or HCV, indicating that special measures should be taken target this group with voluntary and confidential prevention, harm reduction and treatment services.

### Harm reduction in prisons

NSPs, overdose prevention programmes and condoms are not currently available in Italian prisons. OST is provided through public drug dependence service units (SerTs), which receive legislated public funding. SerTs have contracts with 80 % of prison facilities, while the remaining 20 % work on a demand or request basis. Unfortunately, no data is currently being collected on the effectiveness of the service, which is not as widespread as it could be due to a lack of support among politicians and prison authorities. Generally speaking, admitting to the need for harm reduction services in prisons is considered an acknowledgement that existing programmes in the broader community have failed.

## Latvia^12^

### Legal/policy context

The right to health is enshrined in article 111 of the Latvian Constitution, which provides that the State must protect health and guarantee a basic level of medical assistance for everyone [42]. Unfortunately, this remains difficult to uphold in practice because Latvia’s health system has one of the lowest levels of funding in the EU [43].

Drug use in Latvia is charged as an administrative offence, punished by a warning or a fine of up to 280 euros, though repeat use within 1 year is a criminal offence. Similarly, drug possession for personal use may be charged as a criminal or administrative offence depending on the quantity. People with a drug dependency may be exempted from punishment if they voluntarily agree to undergo treatment.

Harm reduction is supported in Latvia. The first NSP began operating in 1997, and today, a network of low-threshold centres for people who use drugs operate throughout the country, providing NSPs, outreach, voluntary HIV testing and counselling, HCV testing, disinfectants, condoms and harm reduction information and education [44]. OST has been available since 1996, and the service is now fairly widely available thanks to a change to the legal framework in 2012 requiring broader provision through general physicians who have completed special training. The regulation also provided for continuity of OST provision in prison. Methadone is provided free of charge, while buprenorphine is available at the patient’s expense. While these changes contributed to a threefold increase in the number of people receiving OST between 2006 and 2012, Latvian coverage rates are still the lowest in the EU [45]. Following the financial crisis in 2009, national structural reforms further impacted the availability and quality of existing harm reduction services. Several agencies working on harm reduction were closed and funding for NGO-run programmes became even more limited [46].

### Prison health

Prison health care is separated from the general health care system in Latvia. Although HIV and TB prevention and treatment in prisons are funded under the health budget as part of a national programme run by the Ministry of Health, prison health care is organised and funded by the Ministry of Justice and all prison health care services are provided by the Latvian Prison Administration [47]. As a result of the economic crisis and cuts to public spending, Latvia’s prison budget shrank by 26 % in 2010 [48], which has inevitably had an impact on the prison health service.

Latvia has 11 prisons and 4745 prisoners, including pre-trial detainees. The prison population rate, a staggering 239 per 100,000 inhabitants, remains one of the highest in the EU. In 2014, according to the Latvian Prison Administration, 618 prisoners self-identified as drug users, while 1059 cases of drug use within prisons were recorded, 763 (72 %) of which were through injection [49].

There is no uniform procedure for health care reporting and data collection in Latvian prisons [50], which means that existing data on HIV, HCV and TB prevalence vary quite significantly and are not very reliable. Data provided by the prison administration, however, suggests that in 2013, 13 % of prisoners were living with HIV, while 34 % were living with HCV [51]. Despite the discrepancies in data and a lack of disaggregation, prevalence rates, as well as the risk of infection, remain much higher in prisons than in the broader community [52]. In 2011, the European Committee on the Prevention of Torture noted that, despite the large proportion of prisoners living with HIV, extremely limited arrangements had been made to provide for their appropriate care. In particular, a very small number of these prisoners were receiving antiretroviral therapy (e.g. three out of 47 inmates at Jelgava Prison; four out of 68 at Valmiera Prison). Furthermore, it appeared that no information on HIV, HCV and prevention methods was made available to staff and prisoners [53]. In addition, general screening for HCV is not provided in prisons; only when prisoners show symptoms can they be tested.

### Harm reduction in prisons

Although harm reduction implementation appears in many prison policies and strategies in relation to health and drugs, harm reduction in Latvian prisons primarily takes the form of information and education campaigns, which are largely provided by the Centre for Disease Prevention and Control and various national NGOs. The provision of free condoms is considered insufficient, there is no NSP provision, and OST (methadone) is only available to prisoners who were receiving it prior to incarceration because there are no medical professionals qualified to provide the service within prisons. In 2014, 28 prisoners were able to continue OST upon incarceration, up from 11 the previous year.^13^

According to civil society, harm reduction services are very difficult to implement in Latvian prisons at the moment due to a shortage of financial and human resources for health, inadequate prison infrastructure, and a lack of education on drugs and health among prison employees. There are also serious reservations regarding the provision of NSPs. Essentially, experts in the country believe that making NSPs available in prisons would implicitly acknowledge the availability, and condone the use, of drugs in prison settings, thereby undercutting the objectives of drug prohibition [54].

## Poland^14^

### Legal/policy context

Poland’s 1997 Constitution protects the right to health, including access to health services, and imposes positive obligations on state authorities to combat the spread of epidemics [55]. The country’s drug laws and policies, however, are some of the most restrictive in the EU. The use, possession, production and trafficking of drugs are all prohibited. The possession of even a small amount of drugs carries a 3-year prison sentence [56]. In 2011, the law was amended (clause 62a) to allow for the suspension of criminal proceedings for possession of drugs intended only for personal use. This flexibility clause, however, has not been applied evenly across the country due to a lack of training on it. In 2013, only around 25 % of all drug possession cases were suspended [57].

Harm reduction enjoys some support in Poland. Harm reduction services have been available to some extent since 1989, and consist of NSPs, OST, and the provision of prevention-related information [58]. In 2013, 13 NSPs were operating in 10 cities, generally run by NGOs, but coverage has actually been declining since 2002 due to a variety of factors, including a lack of funding [58]. OST is currently available at 31 different public and private health care facilities throughout the country and, according to national data, between 13 and 26 % of people who use opioids (2061 people) are accessing this service. This percentage, however, does not include prisoners.

### Prison health

The economic crisis led to a 175 million Euro decrease in prison expenditures between 2008 and 2012. This has inevitably had a negative impact on prison health, which is under the authority of the Ministry of Justice. No plans are currently in place to transfer this responsibility over to the Ministry of Health.

There are 150 penitentiary units in Poland, 60 % of which are prisons. As of 24 July 2015, there were 74,234 inmates, 68,000 of which had been convicted.^15^ While there is no data on the number of people incarcerated for drug offences at any given time, according to the Ministry of Justice, over 10,000 people were convicted for drug possession in 2014 [59].

According to the Polish Prison Service, approximately 4000 prisoners are voluntarily tested for HIV and 9000 for HCV every year. Of these, between 30 and 50 new HIV infections and nearly 400 new HCV infections are detected every year [60]. Access to ART in prisons has increased in the last 5 years [60], and currently, every prisoner living with HIV has access to the service [61]. Data relating to HIV in Polish prisons, however, is not considered to be very reliable. Prisoners’ have complained about their HIV status being disclosed to prison officers, leading many to hide their status. There have also been reports of a lack of skilled physicians resulting in long waiting times for specialist medical consultations, as well as the fact that some physicians are also part of the prison service [62].

### Harm reduction in prisons

NSPs and information on safer injecting are not currently available in Polish prisons because authorities believe this would imply support for drug use during incarceration. Of the 31 OST programmes available throughout Poland, seven are operating across 27 prisons. Although methadone is the most common agent prescribed, buprenorphine, suboxone and other substitutes are also available. Since OST became available in 2003, only 468 prisoners have accessed the service, and according to data obtained from the Polish Prison Service, the number of prisoners accessing the service is decreasing every year [63].

According to the Director of Poland’s Harm Reduction Foundation, Magdalena Bartnik, the provision of OST in the country, including in prisons, is problematic. The service pursues the ultimate goal of abstinence and conditions often apply to the actual provision of OST. Those enrolled in the programme are tested for the presence of drugs other than the substitute, and for those not incarcerated, if a test comes back positive, they are unable to take the substitute home. For prisoners, the treatment is not holistic. There is an apparent lack of HIV and HCV prevention, treatment and care and no real provision of social support, including in the process of re-integrating into the broader community. Furthermore, there is no evaluation on the effectiveness of OST in prisons [64]. The European Committee on the Prevention of Torture has said that interviews with prison doctors revealed a high amount of scepticism around the implementation of OST in Polish prisons [65]. The monitoring mechanism has recommended that the Polish government develop and implement a comprehensive policy for the provision of care to prisoners with a drug dependence, though there are still no signs that steps are being taken to this effect.

## Portugal^16^

### Legal and policy context

Portugal’s constitution enshrines the right to health and establishes the country’s National Healthcare Service [66], which is charged with ensuring every citizen’s preventive, curative and rehabilitative medical needs. As is the case in many countries, however, there is significant gap between law and practice, especially in prison contexts. The on-going economic crisis has resulted in serious cutbacks, especially to the public health and prison sectors.

Portugal has supported harm reduction since the 1970s. The first OST programme was initiated in Boavista in 1977, and shortly thereafter the first Centre of Integrated Responses opened, providing treatment, prevention, harm reduction and reintegration services to people who used drugs through multi-disciplinary teams of doctors, nurses, psychologists and social workers [67]. In 1993, a national NSP was implemented in pharmacies across the country. By 1997, a national network of support had been developed for people who use drugs, covering every district and providing a wide range of services, including harm reduction [68]. Despite these efforts, in 1999, Portugal had the highest HIV prevalence rate among people who inject drugs in the EU [69]. The National Strategy to Fight Drugs adopted that same year and overseen by the Ministry of Health, embraced harm reduction as part of an integrated national strategy. A radical policy shift followed in 2001, in which Portugal decriminalised all drug use, as well as purchase and possession of drugs deemed reasonable for personal use. Today, harm reduction in Portugal is coordinated by the government-run General Directorate for Interventions on Addictive Behaviours and Dependencies (*Serviço de Prevenção e Tratamento da Toxicodependência*, SICAD) and consists of free or very inexpensive medical checks; free psychosocial assessments; free, voluntary and confidential blood sample collections; free OST and NSPs; and social support services.

### Prison health

Despite the integration of prison healthcare into the National Healthcare Service in 2007, many health services are still being provided by the prison system and there appears to be a growing trend towards outsourcing prison healthcare to private contractors. Prisoner complaints collected by civil society expose prison health care as being highly ineffective due to a lack of medicine, medical staff, treatment, access to basic diagnostic care and transportation.

There are currently 49 prisons in the country, including one prison hospital. As of 31 December 2014 there were 14,003 prisoners, about 16 % (2217) of which were incarcerated for drug-related offences [70].^17^ Official data on drug use and prevalence rates of HIV and HCV within prisons is scarce due to the absence of an information system on prison health, as well as difficulty in getting prison authorities to collaborate with information requests. A National Inquiry into Addictive Behaviours within Prison recently undertaken revealed that of a 20 % sample of all prisoners from 47 of the 49 prisons, 3.8 % of those questioned identified as being HIV positive, compared to 10 % in 2007 and 16.3 % in 2001 [71]. Of that same sample, 69.1 % reported using drugs at any point during their lives, 47.9 % of which had used drugs in prison. SICAD’s 2014 annual report reveals that of the 1524 prisoners participating in its drug treatment programmes, 15 % were living with HIV and 42 % with HCV. Of the 15 % living with HIV, 76 % were receiving antiretroviral treatment, and 59 % were HCV co-infected [72].

### Harm reduction in prisons

The various National Strategies to Fight Drugs have outlined a series of harm reduction services to be implemented in prisons, which have focused on facilitating access to condoms and bleach, staff training and prevention programmes [67].

Following ten years of fervent opposition from prison security staff unions, in December 2007 a pilot NSP mandated by Parliament ran for six months in two prisons. Unfortunately, despite guarantees of confidentiality, not a single prisoner participated in the programme during that time, leading to the pilot’s termination. A subsequent study revealed that prisoners’ had in fact feared being discriminated against by, and suffering reprisals at the hands of, prison authorities for their participation in the NSP. The same study revealed that 60 % of the staff at the two participating prisons thought this fear was well founded [73]. At present there are no NSPs available in Portuguese prisons, despite continued reports of prisoners sharing injecting equipment during incarceration [71].

Pilot OST programmes, using methadone and funded by the Directorate-General of Prisons services, were initiated in prisons in Lisbon and Porto in the 1990s. Following evaluations that yielded positive results in 1999, two more programmes were launched. OST has since become the most widespread harm reduction service available in the country, and is currently available, at least in principle, in all 49 prisons in the country. A study of a 20 % sample of the prison population revealed that of the 69.1 % who acknowledged having consumed drugs at any point during their lives, 45.6 % have been in a treatment programme^18^ at some point outside of prison; 18.6 % are currently in a treatment programme in prison, and 27.4 % has already been in a treatment programme in prison. Of that same sample, 13.8 % acknowledged having injected drugs outside of prisons, while 1.1 % acknowledged injecting during their incarceration, compared to 20.6 % and 3.1 % respectively in 2007 and 32.3 % and 11.3 % in 2001 [71]. While this certainly demonstrates progress, some problems remain. According to civil society organisation *Groupo de Ativistas em Tratamento* (GAT*)*, OST is occasionally offered according to institution-specific policies, which may not follow national guidelines. Prisoners have also complained of long waiting times and other difficulties in accessing OST while in prisons [74]. Members of SICAD, the only authorised methadone distributer in the country and the body currently funding and providing OST in prisons nationwide, have also privately expressed frustration with barriers to accessing OST in prisons [75].

The same national study on a 20 % sample of the prison population revealed that 79 % of the sample had never used condoms during conjugal visits, while 72.1 % acknowledged having never used condoms in other contexts, suggesting that condoms are either not available or not easily accessible in Portuguese prisons [71].

## Conclusions

As this overview illustrates, the provision of harm reduction in prisons continues to be largely inadequate compared to the progress achieved outside of prisons. All of the countries reviewed provide a wide range of harm reduction services in the broader community, but the majority fail to provide these same services, or the same quality of these services, in prison settings, in clear violation of international human rights law and minimum standards on the treatment of prisoners. Where harm reduction services have been available and easily accessible in prison settings for some time, better health outcomes have been observed, including significantly reduced prevalence and incidence of HIV and HCV.

While there is no blueprint or ‘one size fits all’ approach to implementing harm reduction in prison settings, certain themes and lessons can be drawn from these countries’ experiences that may be instructive for other countries committed to introducing or scaling up harm reduction in prison settings, or useful for anyone advocating for harm reduction scale up in prisons.

First, the implementation and success of harm reduction approaches and services in prisons, as demonstrated by the Catalan experience, appear to be facilitated by an enabling environment with a range of features. To begin with, a supportive legal and policy context is indispensable. This should include, at the very least, the legal recognition and protection of human rights, including the right to health for everyone, the decriminalisation or legalisation of drug possession for personal use and explicit support for harm reduction, including in prisons, in national policies. A strong and well-funded public health system with extensive coverage and within which prison health is truly integrated is also very important. In this regard, prison health should fall under the responsibility of health ministries, as opposed to ministries of justice or interior, to help ensure that prison health policies and services are integrated into, and compatible with, national health policies and services, and to ensure adequate continuity of care upon prison entry and release. While decentralisation of power, including of health services, is great step in protecting the rights and interests of local populations, steps must be taken to ensure the same, high-quality standard of care for everyone, including prisoners. Finally, it appears that prison authorities can have a significant amount of influence over whether or not harm reduction services are provided in prison settings. Increasing efforts at the national level to engage these key actors, including providing quality training on human rights and harm reduction, would likely go a long way in gaining their support.

Another major theme emerging from this overview is the incredible shortage of resources for harm reduction, as well as the vulnerability of harm reduction programmes to funding cuts. Every country in this overview has been heavily impacted by the financial crisis, with national health and prison budgets suffering extensive cuts, and harm reduction programmes—inside and outside of prisons—being discontinued. At the same time, international donors are shifting their funding for harm reduction from middle-income countries to low-income countries, maintaining that these countries can now afford to provide health services without international assistance [1]. This funding crisis for harm reduction, however, is not always a question of a deficiency of resources but often of a misallocation of resources. In those countries with punitive drug laws, a lot of resources end up being spent on ineffective and often damaging drug law enforcement approaches instead of on interventions that could promote the rights, health and dignity of people who use drugs. Financial and human resources for the provision of quality harm reduction services within prison settings must urgently be scaled up to correspond with need, which, as this study confirms, continues to be very high.

This overview also reveals a striking scarcity of data available on health, drug use and harm reduction in prisons. Where data are available, they are often either very difficult to access or conflict with other available data, and are rarely, if ever, disaggregated on any of the prohibited grounds of discrimination, making it difficult to identify gaps and disparities. There is an urgent need for national collection of reliable data in relation to harm reduction, drugs and HIV, HCV and TB in prisons settings to enable quality monitoring and evaluation of the effectiveness of policies and services. This should be undertaken in a transparent, systematic and comprehensive manner and should be made widely available to the public.

Finally, at the service provision level, an important lesson on accessibility and coverage can be distilled from the overview. Above all, just because a service is available does not mean it is accessible. Concrete steps must be taken to ensure greater accessibility. While all countries reviewed provide OST in prisons, accessibility to this service varies considerably and would be enhanced if, as a first step, all conditions and restrictions on eligibility were lifted. For example, OST provision should not be limited to those who were already receiving it prior to incarceration. Nor should it be dependent on passing mandatory drug tests or enjoying good mental health. Nor should it be conditional on one’s ability to understand a certain language or provided as part of an abstinence-based approach for detoxification. These all act as obstacles to access. The less restrictions and conditions there are, and the more harm reduction services are tailored to the specific needs of prisoners, the more accessible they become. In this regard, prisoners should to meaningfully participate in the development, implementation and monitoring and evaluation of prison-based harm reduction policies and programmes. Furthermore, policy makers and prison authorities should consult important guidance on prison-based harm reduction services developed by the World Health Organization, United Nations Office on Drugs and Crime and UNAIDS when developing and implementing harm reduction services in prison settings.^19^

Throughout the EU, the availability and accessibility of quality harm reduction services in prisons is uneven and continues to be inadequate compared to the progress achieved in the broader community. It is imperative this gap be closed by immediately scaling up, and ensuring access to, a wide variety of harm reduction services in prisons. It is imperative to achieving important global targets on HIV, HCV and TB, to fulfilling prisoners’ inalienable human rights and to treating prisoners with respect for their inherent dignity and value as human beings.

## Abbreviations

ART: Antiretroviral therapy
EU: European Union
HCV: Hepatitis C virus
HIV: Human immunodeficiency virus
NGO: Non-governmental organisation
NSP: Needle and syringe programme
OST: Opioid substitution therapy
SerTs: Drug dependence service units in Italy
SICAD: Serviço de Prevenção e Tratamento da Toxicodependência (General Directorate for Interventions on Addictive Behaviours and Dependencies)
TB: Tuberculosis

## Footnotes

1 The term ‘prisoner’ throughout this article refers to all people deprived of their liberty, including pre-trial detainees, while the term ‘prison’ refers to all detention facilities.

2 For more information, please visit Harm Reduction International’s webpage on the project at: http://www.ihra.net/prison-project

3 The original report, co-authored by Cristina Fernández Bessa, Gemma Nicolás Lazo and Gerard Viader Sauret, Improving Prison Conditions by Strengthening the Monitoring of HIV, HCV, TB and Harm Reduction: Mapping Report Catalonia (Spain), (2016), is available at: http://www.ub.edu/ospdh/sites/default/files/documents/publicacions/improving_prison_conditions_ospdh_2016_report.pdf. Most, if not all, of the research in this subsection is derived from the original report, and all references have been reproduced.

4 In 2014, Catalonia’s incarceration rate was 123 prisoners per 100,000 inhabitants.

5 International human rights law prohibits discrimination on the grounds of race, colour, sex, gender, language, religion, political and other opinion, health status, legal status, national or social origin or any other status, such as age and sexual orientation.

6 The original report by PRAKSIS—Programme of Development, Social Support and Medical Cooperation, *Improving Prison Conditions by Strengthening the Monitoring of HIV, HCV, TB and Harm Reduction: Mapping Report Greece,* (2015) is available at http://www.praksis.gr/assets/PrisonProjectReport_Greece.pdf. Most, if not all, of the research in this subsection is derived from the original report and all references have been reproduced.

7 In 2015, Greece’s prison population rate was 109 per 100,000 inhabitants.

8 The original report, co-authored by Catherine MacNamara, Lorraine Varley and Patricia Mannix McNamara, *Improving Prison Conditions by Strengthening the Monitoring of HIV, HCV, TB and Harm Reduction: Mapping Report Ireland,* (2015), is available at http://www.iprt.ie/files/PrisonProjectReport_Ireland_print_A5.pdf. Most, if not all, of the research in this subsection is derived from the original report, and all references have been reproduced.

9 In 2015, Ireland’s prison population rate was 80 per 100,000 inhabitants.

10 The original report, co-authored by Alessio Scandurra, Sandro Libianci and Grazia Parisi, *Improving Prison Conditions by Strengthening the Monitoring of HIV, HCV, TB and Harm Reduction: Mapping Report Italy,* (December 2015), is available at http://www.associazioneantigone.it/upload2/uploads/docs/Report_Italy_ENG.pdf. Most, if not all, of the research in this subsection is derived from the original report and all references have been reproduced.

11 In 2015, Italy’s prison population rate was 86 per 100,000 inhabitants.

12 The original report, co-authored by Anhelita Kamenska (Latvian Centre for Human Rights) and Solvita Olsena (University of Latvia, Faculty of Medicine), *Improving Prison Conditions by Strengthening the Monitoring of HIV, HCV, TB and Harm Reduction: Mapping Report Latvia,* (2015), is available at http://cilvektiesibas.org.lv/en/publications/improving-prison-conditions-by-strengthening-the-m-398/. Most if not all, of the research in this subsection is derived from the original report and all references have been reproduced.

13 Data included in the report of Latvian Prison Administration states that 11 prisoners were provided substitution therapy in 2013; however, in the Final Report regarding implementation of the Programme for the Limitation of Spreading of the Human Immunodeficiency Virus (HIV) Infection for 2009–2013 other numbers are reported.

14 The original report, co-authored by Marcin Wolny and Piotr Kubaszewski, *Improving Prison Conditions by Strengthening the Monitoring of HIV, HCV, TB and Harm Reduction: Mapping Report Poland,* (2015), is available at http://www.hfhr.pl/wp-content/uploads/2016/06/PrisonProjectReport_Poland-1.pdf. Most, if not all, of the research in this subsection is derived from the original report and all references have been reproduced.

15 In 2015, Poland’s incarceration rate was 191 per 100,000 inhabitants.

16 The original report, co-authored by Nuno Henrique Pontes and António Pedro Dores, *Improving Prison Conditions by Strengthening the Monitoring of HIV, HCV, TB and Harm Reduction: Mapping Report Portugal,* (2015), is available at http://home.iscte-iul.pt/~apad/PrisoesEuropa/observatorio/PROJ%20INFECTIOUS%20DISEASE/PrisonProjectReport_Portugal.pdf. Most, if not all, of the research in this subsection is derived from the original report and all references have been reproduced.

17 In 2015, Portugal’s incarceration rate was 138 per 100,000 inhabitants.

18 ‘Treatment programmes’ here refer mainly to OST programmes, but also likely include abstinence programmes and rehabilitation.

19 See, for example, WHO, UNODC, UNAIDS. *Evidence for Action Technical Papers: Interventions to ddress HIV in Prisons - Drug Dependence Treatments*. Geneva: 2007. Available at: https://www.unodc.org/documents/hiv-aids/EVIDENCE%20FOR%20ACTION%202007%20drug_treatment.pdf; UNODC. *A Handbook for starting and managing needle and syringe programmes in prisons and other closed settings.* No date provided. Available at: https://www.unodc.org/documents/hiv-aids/publications/Prisons_and_other_closed_settings/ADV_COPY_NSP_PRISON_AUG_2014.pdf; and WHO, UNODC. *Opioid Substitution Treatment in Custodial Settings: A Practical Guide*. Germany: 2008. Available at: http://www.unodc.org/documents/hiv-aids/OST_in_Custodial_Settings.pdf
